# The influence of stimulus history on directional coding in the monarch butterfly brain

**DOI:** 10.1007/s00359-023-01633-x

**Published:** 2023-04-24

**Authors:** M. Jerome Beetz, Basil el Jundi

**Affiliations:** 1 Zoology II, Biocenter, University of Würzburg, Würzburg, Germany; 2 Animal Physiology, Department of Biology, Norwegian University of Science and Technology, Trondheim, Norway

**Keywords:** Navigation, Dynamic stimuli, Directional coding, Central complex, Vision

## Abstract

The central complex is a brain region in the insect brain that houses a neural network specialized to encode directional information. Directional coding has traditionally been investigated with compass cues that revolve in full rotations and at constant angular velocities around the insect’s head. However, these stimulus conditions do not fully simulate an insect’s sensory perception of compass cues during navigation. In nature, an insect flight is characterized by abrupt changes in moving direction as well as constant changes in velocity. The influence of such varying cue dynamics on compass coding remains unclear. We performed long-term tetrode recordings from the brain of monarch butterflies to study how central complex neurons respond to different stimulus velocities and directions. As these butterflies derive directional information from the sun during migration, we measured the neural response to a virtual sun. The virtual sun was either presented as a spot that appeared at random angular positions or was rotated around the butterfly at different angular velocities and directions. By specifically manipulating the stimulus velocity and trajectory, we dissociated the influence of angular velocity and direction on compass coding. While the angular velocity substantially affected the tuning directedness, the stimulus trajectory influenced the shape of the angular tuning curve. Taken together, our results suggest that the central complex flexibly adjusts its directional coding to the current stimulus dynamics ensuring a precise compass even under highly demanding conditions such as during rapid flight maneuvers.

**Supplementary Information:**

The online version contains supplementary material available at 10.1007/s00359-023-01633-x.

## Introduction

During locomotion, animals face a highly dynamic sensory world. For example, an insect often shows erratic flight maneuvers, which makes the sensory perception of a visual scene highly challenging (Collett and Land 1975; Egelhaaf et al. 2012; Zeil 2012; Boeddeker et al. 2015; Doussot et al. 2021) mainly due to the following two spatio-temporal dynamics: the angular velocity, and the direction (trajectory). Irrespective of the dynamic nature of a visual cue, the insect’s neural system must reliably process directional information at any moment in time (Haberkern et al. 2022). Integrating compass cues over time, especially for long-distance migration, is discussed to compensate for uncertainties arising from noisy compasses (Johnsen et al. 2020). A sensitivity to the stimulus history may therefore be relevant for a successful migration of the monarch butterfly during which some individuals fly up to 5,000 km from the Canadian-US border southwards to their overwintering site in central Mexico (Reppert et al. 2016). To find their way to Mexico, the butterflies use a time-compensated sun compass for orientation (Perez et al. 1997; Mouritsen and Frost 2002; Merlin et al. 2009). Sun compass signals are processed in evolutionarily conserved head direction neurons of the central complex (Heinze et al. 2013; Nguyen et al. 2021, 2022). These neurons have been described in all insects tested so far [locusts (Pegel et al. 2019; Pisokas et al. 2020; Zittrell et al. 2020), bees (Pisokas et al. 2020; Hensgen et al. 2021; Sayre et al. 2021), beetles (el Jundi et al. 2015, 2018), flies (Seelig and Jayaraman 2015; Giraldo et al. 2018; Hulse et al. 2021; Haberkern et al. 2022)]. In addition, the central complex houses neurons that encode the desired direction (Beetz et al. 2022a; Pires et al. 2022), as well as neurons involved in steering (Martin et al. 2015; Beetz et al. 2022a; Matheson et al. 2022), establishing a central hub for goal-directed spatial orientation in the insect brain (Honkanen et al. 2019).

In addition to the sun, many insects are able to detect and use the pattern of polarized skylight for spatial orientation (Homberg et al. 2011). Consequently, many central complex neurons are sensitive to both the sun position and linearly polarized light (Heinze and Reppert 2011; el Jundi et al. 2014; Pegel et al. 2018, 2019; Hardcastle et al. 2021; Nguyen et al. 2022; Takahashi et al. 2022). Directional coding of central complex neurons has traditionally been tested with a polarizer or a virtual sun being rotated at constant angular velocities around the insect’s head (Vitzthum et al. 2002; Heinze and Homberg 2007; Heinze and Reppert 2011; el Jundi et al. 2015; Zittrell et al. 2020; Nguyen et al. 2021, 2022; Beck et al. 2023). However, considering that insects often change their velocity and direction during flight, these stimulus dynamics are highly artificial. The use of virtual realities in which the visual scene is actively controlled by the insect’s intended steering, either walking or flying (Haberkern et al. 2019; Kaushik et al. 2020), allows scientists to study directional coding under more naturalistic stimulus conditions (Seelig and Jayaraman 2015; Green et al. 2017; Turner-Evans et al. 2017, 2020; Fisher et al. 2019, 2022; Kim et al. 2019; Haberkern et al. 2022; Lu et al. 2022; Lyu et al. 2022). However, as the stimulus dynamics in such closed-loop experiments are controlled by both the animal’s trajectory and velocity, it is challenging to dissociate the influence of each parameter on compass coding. Here, we performed tetrode recordings from the central complex of restrained monarch butterflies that were presented with a virtual sun moving at different directions and velocities around the butterfly’s head. By selectively controlling the stimulus’ angular velocity and trajectory, we show that both stimulus velocity and trajectory affects the spatial tuning of central complex neurons.

## Methods

### Animals

Monarch butterflies (*Danaus plexippus*) were ordered as pupae from Costa Rica Entomology Supply (butterflyfarm.co.cr) and kept in an incubator (HPP 110 and HPP 749, Memmert GmbH + Co. KG, Schwabach, Germany) at 25 °C, 80% relative humidity and 12:12 light/dark-cycle conditions. After eclosion, adult butterflies were transferred to another incubator (I-30VL, Percival Scientific, Perry, IA, USA) at 25 °C and 12:12 light/dark condition. Adults had access to 15% sucrose diluted in water ad libitum.

### Visual stimuli

Monarch butterflies were horizontally fixated to a holder and placed at the center of a cylindric arena. To avoid influences of proprioceptive feedback on neural tuning, we restrained the wing and leg movements of the butterflies. The inner diameter and height of the arena were 32 cm and 12 cm, respectively. The arena’s upper inner circumference, at an elevation of ~ 30° relative to the butterfly, was equipped with 144 RGB-LEDs (Adafruit NeoPixel, Adafruit Industries, New York, New York, USA). One of these LEDs provided a single green light spot that served as a virtual sun stimulus (1.74 × 10^13^ photons/cm^2^/s and 1.2° angular extent at the butterfly’s eyes, as measured at the center of the arena). The angular position of the virtual sun was controlled by the Arduino MEGA 2560. To present a stimulus similar to the one used in former intracellular recordings (Heinze and Reppert 2011; el Jundi et al. 2015; Nguyen et al. 2021), a green light spot was revolved clockwise and counterclockwise in full 360° rotations around the butterfly (continuous stimulus). Angular velocities were kept constant but varied pseudo-randomly between rotations (20, 30, 40, 50, 60, 70, 80°/s). An interstimulus time interval of 1 s was set between each angular velocity. In the stationary stimulus condition, the virtual sun was displaced pseudo-randomly to a different angular position every 2 s. During this time, the virtual sun was presented once at each of the 144 possible angular positions. Many central complex neurons showed a strong onset response when the virtual sun appeared at the new angular position. To avoid the influence of such onset responses on computing the neuron’s angular tuning, we measured the neural firing rate 20 ms after stimulus appearance. To present a more naturalistic stimulus trajectory, the virtual sun was rotated erratically by changing the rotational direction at unpredictable time points. The virtual sun was either moved at constant angular velocities of 30°/s (erratic 30°/s) or 60°/s (erratic 60°/s), or at pseudorandom angular velocities ranging between 20–80°/s in 10°/s steps (erratic 20–80°/s stimulus). An interstimulus-time interval of at least 30 s was set between each condition (erratic 30°/s; erratic 60°/s; erratic 20–80°/s). The unpredictable trajectory was conceptualized so that the virtual sun passed each 10° bin along the azimuth several times at different velocities (for the erratic 20–80°/s stimulus) and angular directions, i.e., clockwise and counterclockwise. Angular velocities between 20–80°/s reflect the most abundant velocities shown by tethered monarch butterflies flying in a flight simulator (Franzke et al. 2020). The order of stimulus presentation (continuous’, ‘stationary’, ‘erratic 30°/s’, ‘erratic 60°/s’, and ‘erratic 20–80°/s’) was random.

### Neural recordings

For neural recordings from 23 monarch butterflies, we custom-built tetrodes and implanted one tetrode in the central complex of each animal. The tetrode consisted of a bundle of five electrodes, four recording and one differential electrode, made from 18 cm-long and 12.5 µm-thin copper wires (P155, Elektrisola, Reichshof-Eckenhagen, Germany). The tetrode was carefully threaded through two Pebax® tubes (each 2–4 cm in length; 0.026’ inner diameter; Zeus Inc, Orangeburg, SC, USA), which served as anchoring points to reversibly mount the tetrodes to a glass capillary. An additional copper wire served as a grounding electrode and was implanted into the posterior regions of the head capsule. All copper wires were soldered to gold pins and attached to an electrode interface board (EIB-18; Neuralynx Inc., Bozeman, MT, USA). Before each experiment, electrode resistances were measured with a nanoZ (Multi Channel Systems MCS GmbH, Reutlingen, Germany) and the electrode tips plated (Elektrolyt Gold solution, Conrad Electronic SE, Hirschau, Germany) to reduce the resistance of each electrode to ~ 0.1–1 MΩ. Tetrodes were reused over multiple experiments; after each experiment the tips were carefully trimmed and replated to the desired resistance.

Prior to obtaining neural signals of central complex neurons, a monarch butterfly was horizontally restrained to a magnetic holder. To minimize movement artifacts during the recordings, the butterfly’s head was waxed to the thorax. The head capsule was opened dorsally and fat and trachea covering the brain surface were removed. To gain access to the central complex, the neural sheath on the dorsal brain surface was carefully removed using fine tweezers. Tetrode tips were immersed in ALEXA 647 Hydrazide (A20502 diluted in 0.5 M KCl, Thermo Fisher Scientific GmbH, Dreieich, Germany) or ALEXA 568 Hydrazide (A10437, Thermo Fisher Scientific GmbH, Dreieich, Germany) to determine the tetrode position after each experiment. The tetrode, together with the glass capillary, was attached to an electrode holder (M3301EH; WPI, Sarasota, FL, USA) and its position controlled via a micromanipulator (Sensapex, Oulu, Finland). After adjusting the tetrode position along x- and y-axes, hemolymph fluid covering the brain was temporarily removed and the tetrode was carefully moved along the z-axis to reach the central complex. While moving along the z-axis, band-pass filtered (600–6,000 Hz) neural signals were measured at a sampling frequency of 30 kHz. Neural signals were sent from the EIB-18 via an adapter board (ADPT-DUAL-HS-DRS; Neuralynx Inc., Bozeman, MT, USA) to a Neuralynx recording system (DL 4SX 32ch System, Neuralynx Inc., Bozeman, MT, USA). Neural activity was monitored using the software Cheetah (Neuralynx Inc., Bozeman, MT, USA). For setting a differential configuration, one electrode of the tetrode was set as a reference for the recording electrodes. To find visual neurons in depths between 150 and 450 µm, the virtual sun was occasionally revolved around the insect’s head in clockwise and counterclockwise directions at an angular velocity of 60°/s.

### Visualization of electrode tracks

After recordings, the brain was dissected out of the head and fixed overnight in 4% formaldehyde at 4 °C. The brain was then transferred into a sodium-phosphate buffer and rinsed for 2 × 20 min in 0.1 M phosphate buffered saline (PBS) and 3 × 20 min in PBS with 0.3% Triton-X. The brain was dehydrated with an ascending ethanol series (30%—100%, 15 min each) and immersed in a 1:1 ethanol/methyl salicylate solution for 15 min, followed by a clearing step in 100% methyl salicylate for at least 1 h. It was mounted in Permount (Fisher Scientific GmbH, Schwerte, Germany) between two cover slips and scanned with a confocal microscope (Leica TCS SP2, Wetzlar, Germany) using a 10 × water immersion objective (HCX PL-Apo 10x/0.4 CS, Leica, Wetzlar, Germany). To visualize the tetrode positions, we reconstructed the tetrode tracks from different experiments in 3D using the software Amira 5.3.3 (ThermoFisher, Germany). To compare tetrode positions from different experiments, we warped each 3D-reconstructed tetrode into the monarch butterfly standard central complex (Heinze et al. 2013). We used an affine (12-degrees of freedom), followed by an elastic registration to transfer the neuropils of the individual central complexes into the corresponding neuropils of the standard central complex. The registration and deformation parameters were then applied to the tetrode reconstructions to visualize the tetrodes in one frame of reference.

### Spike sorting and spike shape analysis

Neural recordings were spike sorted with the tetrode configuration implemented in Spike2 (version 9.00, Cambridge Electronic Devices, Cambridge, UK). We used two spike detection thresholds (one upper and one lower thresholds; Fig. S1). The time window for template detection was set to 1.6 ms. After spike sorting, a principal component analysis (PCA) was used to evaluate and redefine spike clusters. Spike2 channels were exported as down-sampled Matlab files (3 kHz) and the remaining analysis was done with custom written scripts in MATLAB (Version R2021a, MathWorks, Natick, MA, USA). To visualize spike shapes, the WaveMark channels containing the spike waveforms were additionally exported as non-down-sampled Matlab files (30 kHz).

### Analysis

Circular plots summarizing the neuron’s firing rate across different stimulus positions were computed with the CircHist (Zittrell et al. 2020) and CircStats toolbox for MATLAB (Berens 2009). Circular plots were calculated in response to the continuously rotating virtual sun by assigning each spike to the corresponding 10° bin of the sun’s position. To compare the angular tuning measured with differently lasting stimuli, e.g., 6 s for 60°/s or 12 s for 30°/s, the number of spikes per bin were calculated as events/s (Hz). Since the virtual sun was unequally represented along the azimuth, although we aimed for a homogenous directional representation, angular tuning in response to the erratically moving virtual sun was quantified by calculating the neuron’s median firing rate at each 10° bin.

The directional coding of 147 neurons was analyzed in response to each stimulus paradigm (*n* = 11), i.e., stationary, continuous at seven different angular velocities, erratic at three different velocity modes. Angular sensitivity was quantified by testing whether the circular plots differed from a uniform distribution [Rayleigh test; significance level α = 0.05; CircStat toolbox for MATLAB (Berens 2009)]. This was the case for 90 neurons tested in response to the “stationary” and “continuous” stimulus. 55 and 42 neurons showed angular tunings in response to both a continuously and erratically moving virtual sun rotating at 60 and 30°/s, respectively. 53 out of 147 neurons were sensitive to the erratically moving virtual sun rotating at varying velocities (erratic 20–80°/s) and to the continuously moving virtual sun. 45 out of 147 neurons were sensitive to the erratically moving virtual sun irrespective of the angular velocity. In the case of angular sensitivity, we calculated the mean vector, or preferred firing direction (*pfd*) of a neuron, for each stimulus condition. The tuning directedness was calculated as the length of the mean vector (*r*), which could range from 0 (non-directed) to 1 (highly directed). Heatmaps representing the normalized firing rate as a function of virtual sun position were computed to visualize angular tuning of a population of neurons. Angular tuning in response to different stimulus paradigms was statistically compared by considering three different parameters, the neuron’s pfd, tuning directedness, and the tuning shape, represented by the circular plots. Pfds were statistically compared by computing the circular distance between pfds and testing whether the circular distance clustered around 0° (V-test: 0° expected), indicating that the pfds resembled each other across the considered stimulus paradigms. To compare pfds across more than two stimulus paradigms, we computed the circular variance of pfds and statistically compared them against shuffled pfds (Mann–Whitney test). For shuffling the pfds, we randomly considered pfds from different neurons. For a more detailed comparison of angular tuning, we correlated the angular tuning measured in response to different stimulus protocols and compared the correlation values with a Kruskal–Wallis test + Dunn’s multiple comparisons test. Tuning directedness measured in response to different stimulus paradigms was statistically compared with a non-parametric Wilcoxon matched-pairs signed rank test (comparison of two groups) or with a non-parametric Friedman test (comparison of multiple groups). Based on the tuning directedness as a function of angular velocities, we categorized the neurons into four different groups, low pass, high pass, band pass, and multi-peaked tuning curves. We calculated a neuron-specific threshold (*thv*) with the following equation:

$$thv={r}_{max}-\frac{({r}_{max}-{r}_{min})}{2}$$

where r_max_ represents the neuron’s maximum vector length and r_min_ the neuron’s minimum vector length.

Respectively, low- and high-pass neurons passed the threshold only at low and high angular velocities, respectively. Band pass neurons passed the threshold at a particular range of angular velocities, while their tuning directedness dropped below the threshold at low and high angular velocities. Multi-peaked neurons showed a notch, i.e., a drop below the threshold, at certain angular velocities, while angular velocities below and above this notch resulted in tuning directedness above the threshold. For each neuron, we also computed the “best velocity” representing the angular velocity that induced the neuron’s maximum tuning directedness.

Based on the neural responses measured with the predictably moving virtual sun, we modelled for each neuron the angular tuning to the erratically moving virtual sun at varying velocities. This modelled angular tuning was then compared with the measured one. To model the response to the erratic stimulus, we first split the stimulus into 36 bins, each covering 10°. We then counted how often and at which angular velocity the virtual sun traversed each bin. For instance, the 10° sector between 145° and 155° was passed by the virtual sun in total 15 times with different angular velocities as follows: two times with 20°/s, two times with 30°/s, two times with 40°/s, once with 50°/s, three times with 60°/s, three times with 70°/s, and two times with 80°/s. We took the firing rate measured at this 10° bin at different angular velocities when the virtual sun was continuously revolved around the butterfly and weighted them accordingly to the erratic stimulus trajectory. This process was done for each 10° bin to predict an angular tuning to the erratically moving virtual sun.

### Statistics

Circular statistics were performed with the CircStat toolbox for MATLAB (Berens 2009) and Oriana (Version 4.01, Kovach Computing Services, Anglesey, Wales, UK). All linear statistics were computed in GraphPad Prism 9 (GraphPad Software, San Diego, CA, USA). Sample sizes were not statistically pre-determined. Data distributions were tested for normality with a Shapiro–Wilk test. Normally distributed data were further analyzed with parametric statistical tests, while non-normally distributed data were tested with non-parametric tests. Statistical tests were always two-sided. Data collection and analysis were not conducted blind to the conditions of the experiments. For neural recordings, stimulus presentation was pseudorandomized.

## Results

### Increase in stimulus velocity sharpens angular tuning in the central complex

**Fig. 1:**
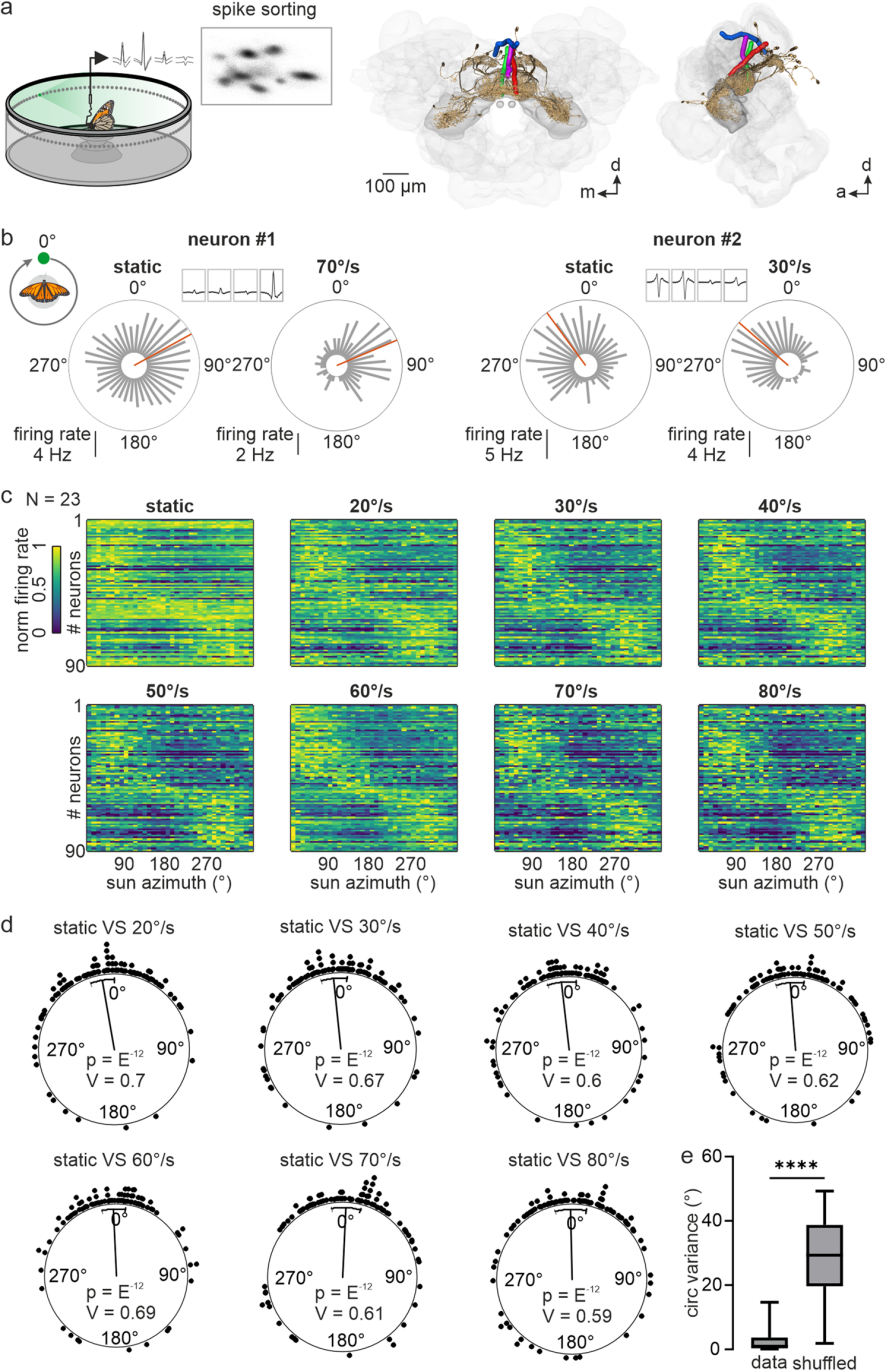
Influence of the stimulus’ angular velocity on directional coding in the monarch butterfly central complex. **a** (left) Schematic of the setup and exemplary principal component analysis used for spike sorting. (right) Anterodorsal and lateral view of reconstructed tetrode tracks from four animals warped into the standardized monarch butterfly central complex. Prominent central complex neurons (brown) are illustrated to visualize neural fibers entering the central complex. **b** Comparison of the angular tuning of two representative neurons measured with a stationary virtual sun with its position changed randomly every 2 s (static) and with a virtual sun revolving in full rotations around the animal. Preferred firing directions (*pfds*) are indicated by red lines. During all recordings, the butterfly’s head faced 0°. **c** Comparison of directional coding of 90 neurons recorded in 23 animals in response to a stationary and to a continuously rotating virtual sun at different angular velocities. Each line represents the angular tuning of one neuron. **d** Comparison of the pfds measured in response to the stationary and rotating virtual sun. Each dot represents the difference in the pfd of one neuron. Values clustered around 0° (V-test: *p* < 0.05) indicate that the pfds did not differ significantly across the neural population (n = 90). **e** Circular variance of the pfds within a neuron was lower than the circular variance calculated with shuffled data, i.e., comparing pfds across randomly selected neurons (Mann -Whitney test: *p* < 10^–5^; *n* = 90; *U* = 306)

To investigate the influence of stimulus dynamics on directional coding (Fig. 1a), we performed long-term tetrode recordings from the central complex of 23 restrained monarch butterflies. While the butterflies were placed at the center of a cylindrical arena, a green light spot, representing a virtual sun, was presented to the animals from different azimuthal positions. The virtual sun was either randomly displaced to different azimuthal positions every 2 s (*static stimulus*) or was moved on circular paths around the butterfly at different, constant angular velocities (20°-80°). We measured the angular tuning of 90 spike-sorted single units (Fig. S1; 4 ± 2.5 units/animal; *N* = 23), from here on referred to as neurons, by calculating the neurons’ mean firing rate at different positions of the virtual sun. Independent of stimulus dynamics, i.e., stationary or rotating, neurons reliably exhibited a maximum spiking activity at a certain position of the virtual sun, from here on referred to as preferred firing direction (pfd, Fig. 1b, red lines in circular plots; Rayleigh test *p* < *0.05*). Different pfds were observed across the neural population (Fig. 1c). The neurons’ pfds were biased toward the frontal visual field and clustered at around -/ + 45°, which is in line with recent findings from the central complex in butterflies (Beetz et al. 2022b; Nguyen et al. 2022) and fruit flies (Seelig and Jayaraman 2013; Fisher et al. 2019). Neurons of the present study showed consistent pfds, irrespective of the stimulus dynamics (V-test expected at 0°: *p* < 10^–12^; *n* = 90; V = 0.7 _stationary vs 20°/s_; V = 0.67 _stationary vs 30°/s_; V = 0.6 _stationary vs 40°/s_; V = 0.62 _stationary vs 50°/s_; V = 0.69 _stationary vs 60°/s_; V = 0.61 _stationary vs 70°/s_; V = 0.59 _stationary vs 80°/s_; Fig. 1d). Pfd stability across different angular velocities was also reflected by a lower circular variance of pfds compared to the variance calculated for shuffled pfds (Mann–Whitney test: *p* < 10^–5^; *n* = 90; *U* = 306; Fig. 1e).

**Fig. 2:**
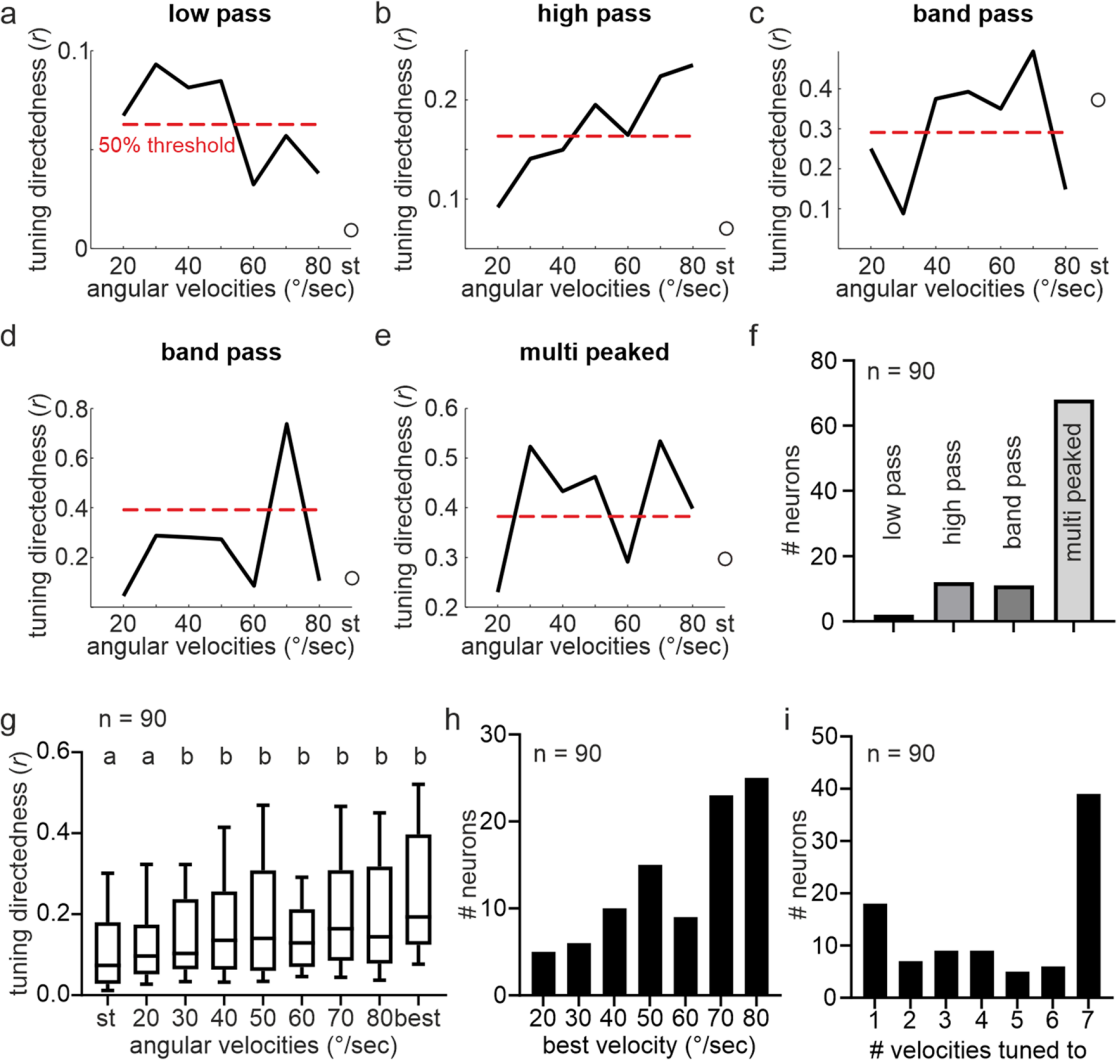
Comparison of tuning directedness measured with a stationary and a virtual sun rotating at different angular velocities. **a-e** Tuning curves of five representative example neurons, demonstrating the diversity of tuning directedness as a function of angular velocity. Dashed red horizontal lines depict the neuron’s specific 50% threshold of tuning directedness. Unfilled dots represent the tuning directedness measured in response to a stationary virtual sun (*st*) that was randomly displaced. Note, that for four of the neurons, the tuning directedness in response to the stationary virtual sun was lower than to the dynamic virtual sun. **f** Histogram summarizes the number of neurons categorized into the corresponding tuning curves, low pass, high pass, band pass, multi peaked. **g** Tuning directedness as a function of angular velocities across the neuronal population (*n* = 90). Letters above boxplots depict the categories described by statistics (Friedman test multiple comparisons: *p* < 10^–5^, *n* = 90; Friedman statistics = 286.6). *st* = stationary; *best* = best velocity, i.e., neuron specific velocity resulting in the highest tuning directedness. **h** Distribution of the velocities that evoked the highest tuning directedness (best velocity) across the recorded neural population. **i** Histograms of the number of angular velocities at which the neurons were tuned to (Rayleigh test: *p* < 0.05)

Although the pfds were unaffected by stimulus dynamics, the neurons seemed to be more sharply tuned to the moving virtual sun than to the stationary virtual sun (Fig. 1c). We quantified the tuning directedness by calculating for each neuron the vector length (*r*) at different angular velocities. We also calculated for each neuron a 50% threshold (c.f. methods) that allowed us to categorize the recorded neurons into 4 groups: low-pass, high-pass, band-pass, and multi-peaked neurons (Fig. 2a-f). Most neurons, 68 out of 90 (~ 76%) showed multi-peaked tuning curves (Fig. 2e, f) as their tuning directedness was high at most of the tested angular velocities but dropped below the 50% threshold at some angular velocities. In twelve neurons (13%), the tuning directedness increased with increasing angular velocities (high pass; Fig. 2b, f). Eleven neurons (12%), categorized as band pass, showed a tuning directedness above the 50% threshold in a particular velocity range (Fig. 2c), or just at one angular velocity (Fig. 2d, f). Only two neurons showed a high tuning directedness at low angular velocities (low pass; Fig. 2a, f). Overall, the tuning directedness to the virtual sun was higher when the stimulus was rotated with an angular velocity of at least 30°/s compared to lower velocities or the stationary stimulus (Friedman test multiple comparisons: *p* < 10^–5^, *n* = 90; Friedman statistics = 286.6; Fig. 2g). In general, the tuning directedness increased with the angular velocities (best velocity; Fig. 2h), with 47 neurons (~ 52%) showing a maximum tuning directedness at angular velocities higher than 60°/s. Only 39 out of 90 neurons (~ 43%) were tuned to all tested angular velocities (Rayleigh statistics p < 0.05) and 18 neurons (20%) were sensitive to only one angular velocity (Fig. 2i) suggesting that the angular velocity had a relatively strong impact on the neuron’s tuning directedness. Taken together, while the stimulus dynamics did not affect the neurons’ pfds, the angular tuning was sharper in response to a moving virtual sun than to a stationary virtual sun. The sharpening in angular tuning increased with increasing angular velocities.

## Influence of stimulus trajectory on neural tuning

**Fig. 3:**
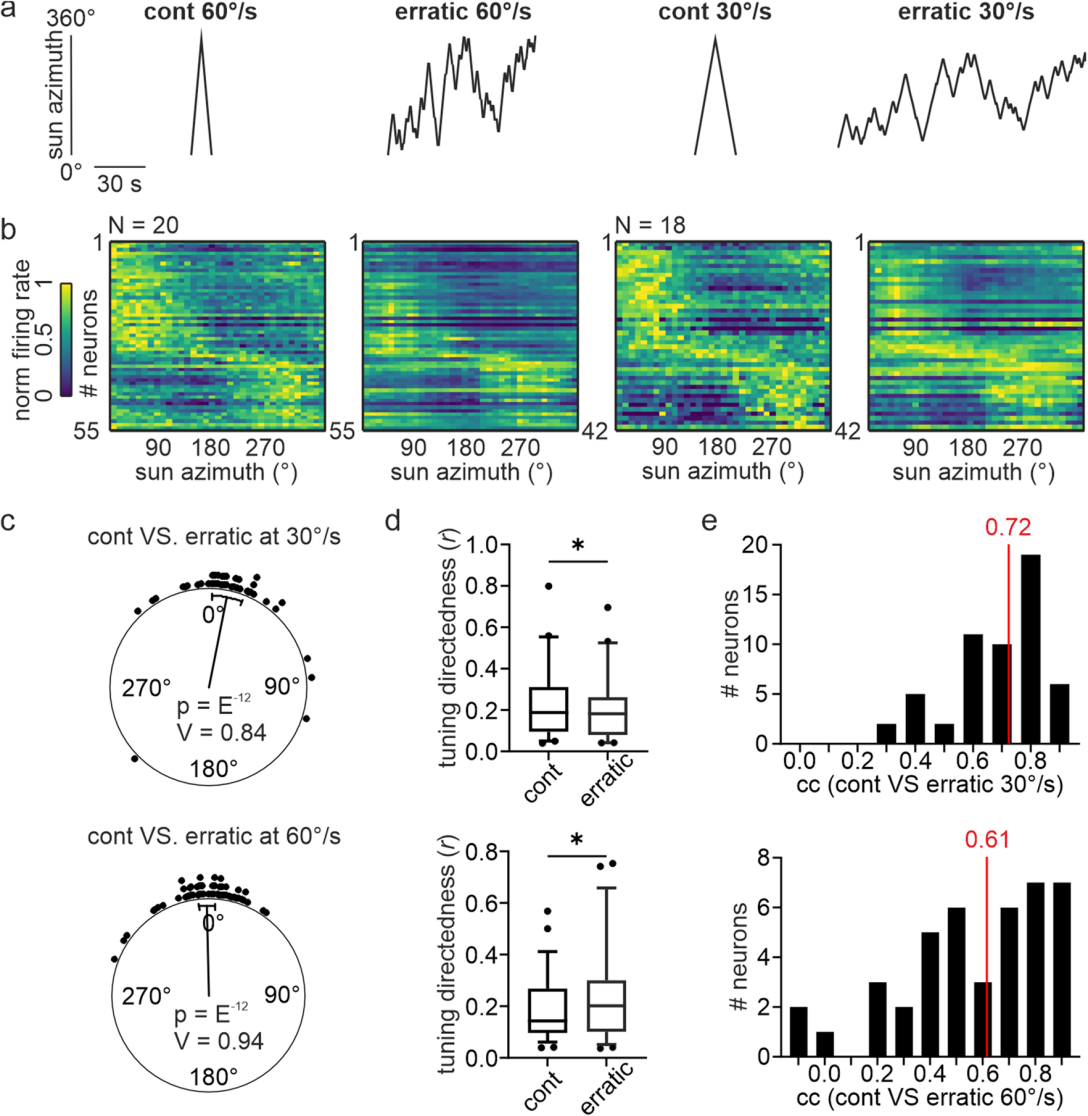
Influence of stimulus trajectory on directional coding. **a** Stimulus trajectories used to measure the angular tuning. *cont* = full rotations, *erratic* = trajectory with changes in angular direction. **b** Comparison of directional coding measured with a continuously rotating virtual sun (first and third heatmap) and an erratically rotating virtual sun (second and fourth heatmap). 55 and 42 neurons were recorded from 20 and 18 animals, respectively. **c** Comparison of pfds measured with a continuously and an erratically moving virtual sun. Each dot represents the pfd difference of one neuron (upper plot: 30°/s, lower plot: 60°/s). Values clustered around 0° (V-test: *p* < 0.05) indicate that pfds did not depend on the stimulus trajectory. **d** Comparison of tuning directedness measured with a continuously (*cont*) and an erratically (*err*) rotating virtual sun at angular velocities of 30°/s (*upper graph*) and 60°/s (*lower graph*). Wilcoxon matched-pairs signed rank test: p (30°/s) = 0.0122; *n* = 42; p (60°/s) = 0.0183; *n* = 55. **e** Distribution of correlation values (*cc*) computed by comparing the angular tuning measured with a continuously and erratically rotating virtual sun at angular velocities of 30°/s (*upper,* *n* = 42) and 60°/s (*lower,* *n* = 55). Median values of the neural population are depicted in *red*

After demonstrating that stimulus velocity affected tuning directedness, we next asked whether the stimulus trajectory might also affect directional coding. We, therefore, measured the angular tuning of central complex neurons in response to a virtual sun that erratically changed its moving direction from clockwise to counterclockwise (and vice versa, *erratic stimulus*) and compared it to the response of the same neurons to the revolving virtual sun (moved by 360° around the animal without any changes in stimulus direction, *continuous stimulus*; Fig. 3a). To avoid any angular velocity effects on neural tuning, we first kept the angular velocity constant at 30° or 60°/s. 55 (2.75 ± 2 neurons/animal) and 42 (2.33 ± 1.2 neurons/animal) out of 90 neurons showed angular sensitivities when the sun was rotated at 60°/s and 30°/s (Rayleigh test: *p* < 0.05; Fig. 3b), respectively. The stimulus trajectory did not affect the neurons’ pfds indicated by pfd differences across stimulus conditions clustered around 0° (V-test: *p* < 10^–12^; *n* = 55; V = 0.94 _cont 60°/s vs erratic 60°/s_; V-test: *p* < 10^–12^; *n* = 42; V = 0.84 _cont 30°/s vs erratic 30°/s_; Fig. 3c). Depending on the angular velocity, the tuning directedness was differently affected by the stimulus trajectories (Fig. 3d). While the tuning directedness to the erratic stimulus was lower than the directedness to the continuously moving sun stimulus at 30°/s (Wilcoxon matched-pairs signed rank test: *p* = 0.0122; *n* = 42), the opposite was true for the 60°/s stimulations (Wilcoxon matched-pairs signed rank test: *p* = 0.0183; *n* = 55). However, it is noteworthy that the median differences in tuning directedness were relatively low (-0.03 for 30°/s; 0.018 for 60°/s), indicating that these effects were subtle.

To directly compare the neural tuning measured with the continuously and erratically rotating sun, we correlated the angular tuning for each neuron between the two conditions. Interestingly, the tuning of several neurons correlated poorly, which resulted in medians across the neuronal populations of only 0.61 (60°/s) and 0.72 (30°/s) (Fig. 3e). This outcome indicates that the stimulus trajectory, indeed, affected the angular tuning of central complex neurons.

**Fig. 4:**
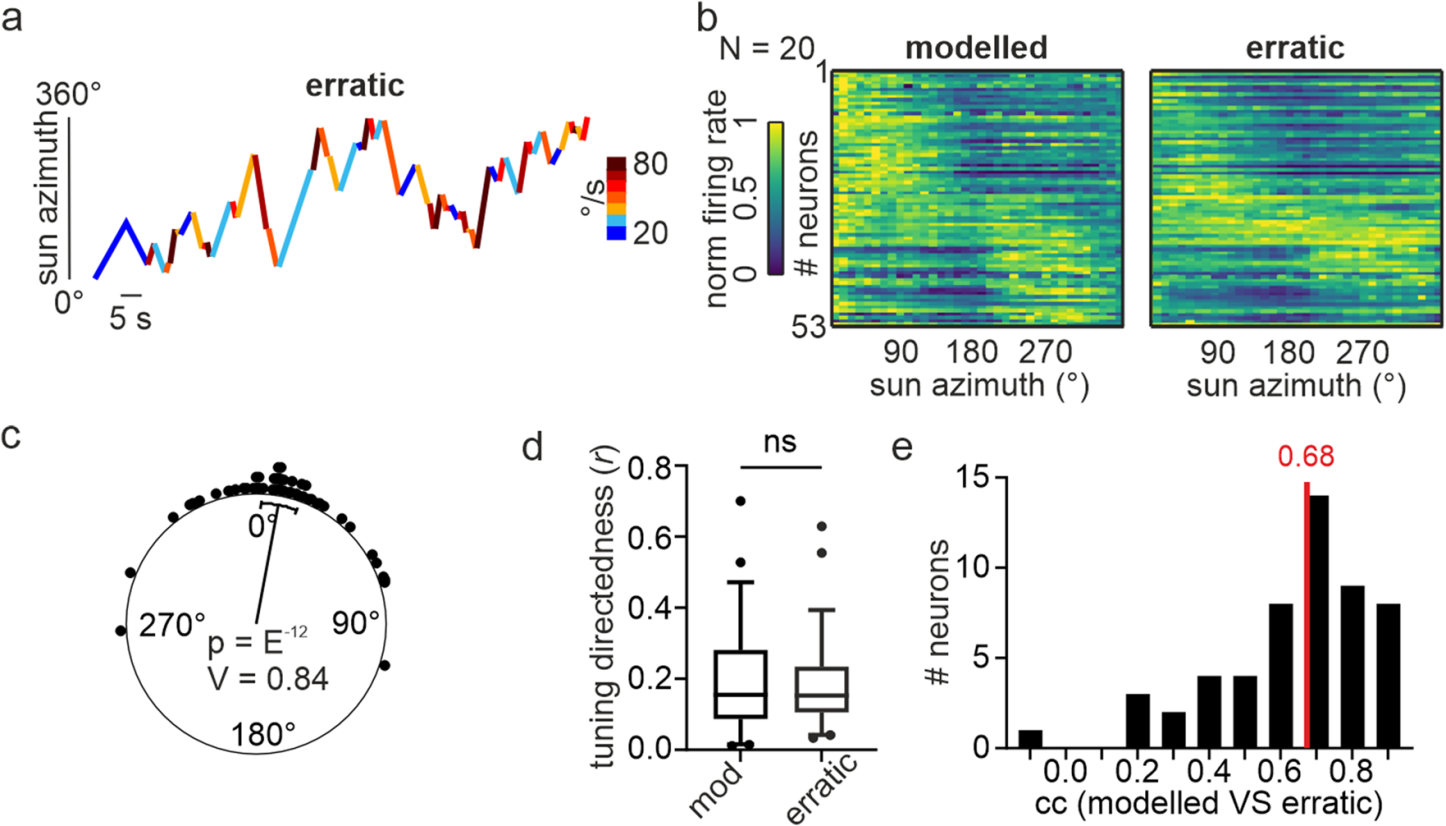
Modelling angular tunings to an erratically moving virtual sun based on neural responses to a continuously rotating virtual sun. **a** Stimulus trajectory of an erratically moving virtual sun. Angular velocity changes after each change in angular direction. **b** Comparison of the modelled angular tuning (left) and the recorded angular tuning (right) measured with the erratic stimulus trajectory. 53 neurons were recorded from 20 animals. **c** Comparison of modelled pfds with the ones measured with an erratically rotating virtual sun. Each dot represents the pfd difference of one neuron. Values clustered around 0° (V-test: *p* < 0.05) indicate that modelled pfds resembled the measured ones. **d** Comparison of modelled (*mod*) tuning directedness with the measured one (*erratic*). Wilcoxon matched-pairs signed rank test: *p* = 0.104; *n* = 53. **e** Distribution of correlation values (*cc*) obtained by comparing the modelled angular tuning with the one measured in response to an erratic trajectory (*erratic*). Median value of the neural population is depicted in *red*

To gain a deeper understanding of the role of the stimulus trajectory on angular tuning, we tested to what extent we could predict the neural response to an erratically moving virtual sun that changed its angular velocity at the same time (20°/s – 80°/s, Fig. 4a). For each neuron (*n* = 53; 2.65 ± 1.9 neurons/animal), we modelled an angular tuning to the erratically rotating virtual sun by considering the responses to the continuously rotating virtual sun, shown in Fig. 1. For example, according to the erratic stimulus trajectory (Fig. 4a), the virtual sun passes the 145° to 155° azimuthal sector 15 times with different angular velocities (two times with 20°/s, two times with 30°/s, two times with 40°/s, once with 50°/s, three times with 60°/s, three times with 70°/s, and two times with 80°/s). Therefore, we expected that the firing rate in this sector would resemble the mean firing rate measured within the same sector when continuously rotating the sun stimulus at these angular velocities. This prediction was computed for each 10° sector around the animal to model a predicted angular tuning for each neuron. We then compared the modelled tuning response of a neuron to the actual recorded response of the same neuron when we presented the erratic stimulus. In this case, the predicted angular tuning resembled the measured one relatively well (Fig. 4b). Thus, the pfds (Fig. 4c; V-test: *p* < 10^–12^; *n* = 53; V = 0.841) and the tuning directedness (Fig. 4d; Wilcoxon matched-pairs signed rank test: *p* = 0.104; *n* = 53) were the same between the modelled and the measured tuning. However, the modelled tuning correlated only weakly (only 0.68) with the recorded tunings (Fig. 4e). These findings further support our working hypothesis that the stimulus trajectory affects the directional coding of central complex neurons.

## Stimulus trajectory affects more than angular velocity directional coding

**Fig. 5:**
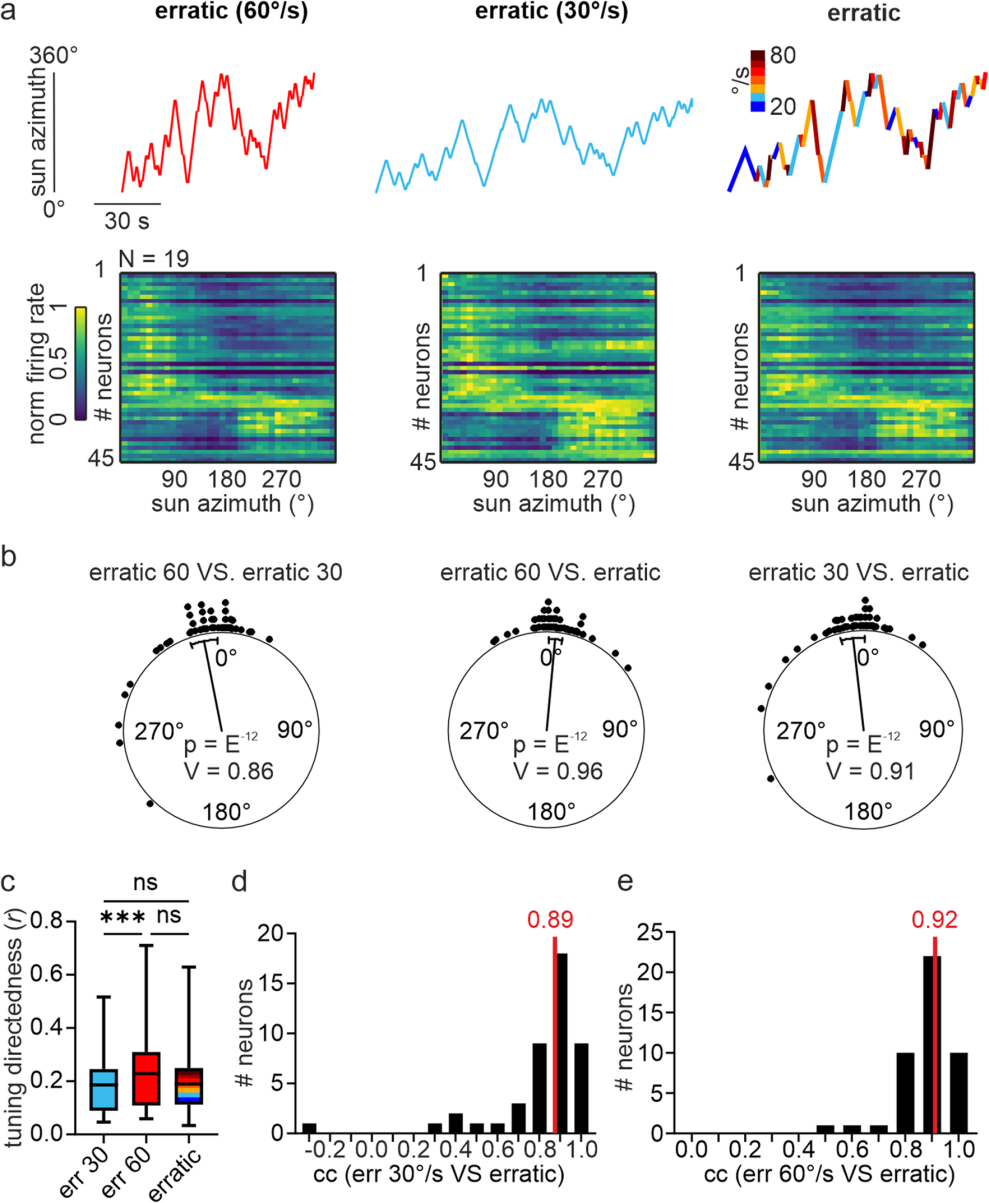
Influence of angular velocity on directional coding. **a** *upper plots*: Stimulus trajectories used to measure the angular tuning. *lower plots*: Comparison of directional coding measured with an erratically moving virtual sun rotating at different angular velocities. 45 neurons were recorded in 19 animals. **b** Comparison of pfds measured with an erratically moving virtual sun rotating at different angular velocities. Each dot represents the pfd difference of one neuron. Values clustered around 0° (V-test: *p* < 0.05) indicate that pfds did not depend on the angular velocity. **c** Comparison of tuning directedness measured with an erratically moving virtual sun rotating at different angular velocities. Friedman test + Dunn’s multiple comparisons test: *p* < 10^–5^; *n* = 45; Z = 4.11 (erratic 30°/s vs erratic 60°/s); *p* = 0.17; *n* = 45; Z = 1.9 (erratic 30°/s vs erratic 20–80°/s); *p* = 0.08; *n* = 45; Z = 2.21 (erratic 60°/s vs erratic 20–80°/s). **d** Distribution of correlation values (*cc*) computed by comparing the angular tuning measured with an erratically moving virtual sun rotating with a constant angular velocity of 30°/s and at varying angular velocities ranging between 20 and 80°/s (*n* = 45). Median cc value is depicted in *red*. **e** The same as **d** but with data from a constant angular velocity of 60°/s and varying velocities ranging between 20 and 80°/s

Next, we examined whether the directional coding was affected more strongly by the stimulus trajectory or by the stimulus velocity. To test this, we measured the angular tuning of 45 neurons (2.4 ± 1.8 neurons/animal) in response to the same stimulus trajectory, i.e., erratic, but at different constant angular velocities, i.e., 60°/s, 30°/s, or at varying angular velocities between 20 and 80°/s (Fig. 5a). The neurons’ pfds did not differ irrespective of which stimulus was presented to the butterflies (V-test: *p* < 10^–12^; *n* = 45; V = 0.86 _erratic 60°/s vs erratic 30°/s_; V = 0.96 _erratic 60°/s vs erratic 20–80°/s_; V = 0.91 _erratic 30°/s vs erratic 20–80°/s_; Fig. 5b), again showing that the spatial tuning is robust to the stimulus trajectory and velocity. The tuning directedness, however, was higher at 60°/s than at 30°/s (Friedman test + Dunn’s multiple comparisons test: *p* < 10^–5^; *n* = 45; Z = 4.11; Fig. 5c), which is in line with the results reported in Fig. 2. The tuning directedness measured with the erratic stimulus and variable velocities was between those measured at constant velocities at 30°/s and 60°/s and was not statistically different (Friedman test + Dunn’s multiple comparisons test: *p* = 0.17; *n* = 45; Z = 1.9 _erratic 30°/s vs erratic 20–80°/s_; *p* = 0.08; *n* = 45; Z = 2.21 _erratic 60°/s vs erratic 20–80°/s_).

**Fig. 6:**
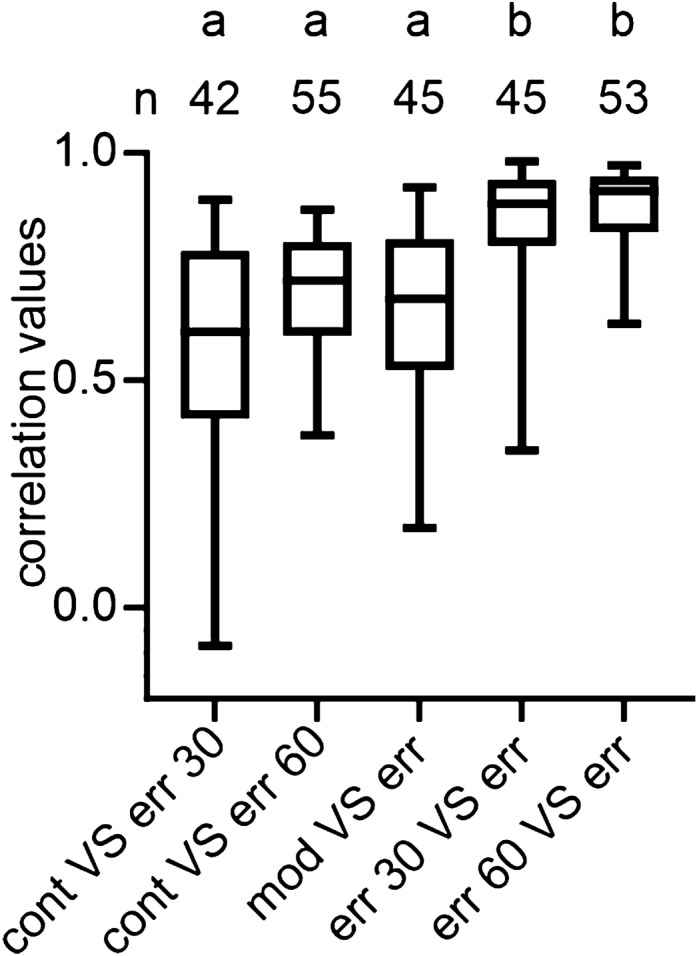
Comparison of correlation values computed by comparing angular tunings measured with different stimulus dynamics. *cont* = continuously rotating virtual sun at 30°/s and 60°/s; *err 30* & *err 60* = erratically rotating virtual sun at 30°/s & 60°/s. *mod* = modelled angular tuning with data based on *cont* at different angular velocities (20–80°/s). *err* = erratically rotating virtual sun at varying velocities (20–80°/s). Kruskal–Wallis test + Dunn’s multiple comparisons test: *p* < 10^–5^

Correlating the angular tunings with each other showed that the directional coding was highly similar between the erratic stimuli in which the virtual sun was moved at constant velocities and the erratic stimulus representing variable angular velocities [median: 0.89 (30°/s erratic stimulus vs. erratic stimulus with changing velocity) and 0.92; (60°/s erratic stimulus vs. erratic stimulus with changing velocity) Fig. 5d, e]. A comparison of angular tunings measured with two different stimulus trajectories, but similar angular velocities (1st, 2nd, and 3rd boxplot in Fig. 6) resulted in lower correlation values than the comparisons of angular tunings measured with a consistent trajectory but at different angular velocities (Kruskal–wallis test + Dunn’s multiple comparisons test: *p* < 10–5; 4th and 5th boxplot in Fig. 6). Our results suggest that the stimulus trajectory mainly affects the shape of the angular tuning curves while the angular velocity mainly affects the tuning directedness (Fig. 2).

## Discussion

Brain activity has often been monitored in response to stimuli that are randomly presented with a fixed interstimulus time interval. Such highly controlled stimulus designs aim to isolate neural responses to single stimuli while avoiding interferences of preceding stimuli on the neural response. However, in nature, cues are usually not processed as discrete events in time but rather form a continuous perception of the cue. Therefore, it is not surprising that many neurons are highly sensitive to the stimulus history and integrate information from subsequent stimuli (Clark and Demb 2016; Weber et al. 2019; Jin and Glickfeld 2020; Benda 2021; López-Jury et al. 2021; Beetz and Hechavarría 2022; Pastyrik and Firzlaff 2022; Price and Gavornik 2022). Here, we explicitly tested how the stimulus history, i.e., angular velocity and trajectory of a compass cue, affects the directional coding in the monarch butterfly brain. Our results show that the directional coding is affected by both the angular velocity and trajectory of a compass cue. A substantial number of neurons increased their tuning directedness when the stimulus was moved instead of being presented stationary at different angular positions. Consistent with findings on compass neurons in *Drosophila* (Turner-Evans et al. 2020), our results suggest that the butterfly’s compass gains precision when the insect performs erratic flight turns (Figs. 1, 2). A similar effect on tuning sharpness has been reported in the mammalian cortex when the animals were stimulated with sequenced stimuli rather than with an isolated stimulus (Wehr and Zador 2005; Beetz and Hechavarría 2022). For example, the spatial tuning of auditory neurons is sharper when bats are stimulated with sequences of echolocation signals rather than temporally isolated acoustic signals (Beetz et al. 2016a, 2017). Sharpening effects may be explained by simultaneous excitatory and inhibitory inputs, with the inhibitory inputs lasting longer than the excitatory ones (Isaacson and Scanziani 2011; Beetz et al. 2016b). Recurrent inhibition is widespread in the insect compass network (Hulse and Jayaraman 2020; Turner-Evans et al. 2020), making it highly likely that neural inhibition is important for the sharpening effect reported here. Intrinsic changes of a cell such as spike-rate adaptation or fatigue have also been discussed as mechanisms for such neural adaptations (Carandini 2000; Benda and Herz 2003; Benda 2021). Intracellular recordings from single central complex neurons are necessary to explicitly test how inhibitory inputs shape specific tuning curves.

While we observed that the angular velocity affected the tuning directedness of central complex neurons, the angular tuning to the virtual sun, represented by the neurons’ pfds, was invariant. Irrespective of whether the virtual sun was displaced to random positions (*static stimulus*) or revolved around the butterflies (*continuous stimulus*), central complex neurons reliably represented the angular position of the virtual sun. This independence of angular tuning on the stimulus dynamics supports the idea that the central complex, as a global compass, needs to operate within different states of locomotion ranging from quiescence to flight (Beetz et al. 2022b). In contrast to this, central complex neurons in the desert locust respond to a small visual cue against a bright background when the cue is moved, but not when it is stationary (Bockhorst and Homberg 2015b). This response rapidly declines when the cue is presented repeatedly (Bockhorst and Homberg 2015b). In monarch butterflies, we did not find any evidence for neural adaptation, i.e., a reduction in neural response to repetitive stimulus presentations. This lack of adaptation cannot be explained by species-specific differences because locust central complex neurons do not show any adaptations to a virtual sun (Takahashi et al. 2022). Therefore, it is likely that the differences in encoding a small moving cue and the virtual sun may arise from differences in the stimulus design, e.g., movement direction, stimulus wavelength. In Bockhorst and Homberg (2015b), the small cues were moved along the translational direction, while we rotated our stimulus around the animals. This indicates that the integration of stimulus history in the central complex may be different between translational and rotational stimulus trajectories (Rosner and Homberg 2013; Bockhorst and Homberg 2015b).

Although the neurons’ pfds were independent of the stimulus dynamics, the stimulus trajectory affected the angular tuning of monarch butterfly central complex neurons. The angular tuning to an erratically rotating virtual sun that moved at different velocities could best be explained when considering the stimulus trajectory (Figs. 5, 6). Stimuli representing different trajectories but moved at similar angular velocities resulted in an angular tuning that was less comparable to the angular tuning measured with an erratically moved virtual sun (Figs. 3, 4). This outcome indicates that directional coding in the central complex is less sensitive to variations in angular velocity than to stimulus trajectories. Sensitivity to the stimulus trajectory is further supported by the observation that the pfds of many neurons in the insect central brain depend on the rotational direction of the stimulus, i.e., whether the stimulus rotates clockwise or counterclockwise (Träger and Homberg 2011; Bockhorst and Homberg 2015a; Beetz et al. 2016b; Stone et al. 2017). The same has also been shown for central complex neurons in cockroaches that were rotated around a stationary visual cue (Varga and Ritzmann 2016). A direction selectivity has also been demonstrated in central complex neurons that transfer rotational optic flow information into the compass network (Green et al. 2017; Turner-Evans et al. 2017; Zittrell et al. 2023).

Future studies will benefit from studying the angular tuning of neurons to a variety of different stimulus trajectories, including naturalistic flight trajectories recorded in flight simulators (Franzke et al. 2020, 2022). Based on our findings in monarch butterflies, it would be informative to test the influence of the insect’s movement history on the directional coding under different virtual reality settings, e.g., open versus closed loop settings.

## Behavioral relevance of encoding stimulus history in the central complex

For goal-directed navigation, animals continuously compare their current heading with their desired goal direction (Dacke and el Jundi 2018; Green et al. 2019; Honkanen et al. 2019). If the current heading deviates from the desired goal direction, animals steer back towards the correct direction. We found that an increase in angular velocity to rotating stimuli results in a higher tuning directedness, suggesting that the precision of the compass increases during goal-directed navigation at higher rotational velocities in monarch butterflies. Whether an increase in translatory velocity also increases the coding precision of the insect’s compass remains to be determined. Finally, future studies should be performed in migratory monarch butterflies to determine if compass precision across various stimulus dynamics might be different, as central complex neurons in their brain are more narrowly tuned to the virtual sun than neurons in non-migratory butterflies (Nguyen et al. 2021).

Because the directional coding of central complex neurons could best be explained by stimulus trajectory, irrespective of varying angular velocity (Fig. 6), this observation suggests that both stimulus position and trajectory are encoded by central complex neurons. Encoding the stimulus trajectory may help to drive moment-to-moment steering decisions (Honkanen et al. 2019). Although this strategy may not be important for long-distance migration, it could be important across smaller spatial scales, such as during central-place foraging. If neurons in the insect central complex can ‘memorize’ cue trajectories, this ability might help them to store a specific route back to their nest based on route-based navigation as exhibited, for instance, by desert ants (Kohler and Wehner 2005; Collett 2010; Pisokas et al. 2022) and bees (Stone et al. 2017; Patel et al. 2022). How exactly this is achieved at the neural level, and if monarch butterflies can make use of such route-based navigation such as during foraging, are exciting questions that will be the focus of future research.

## Supplementary Information

Below is the link to the electronic supplementary material.

Supplementary file1 Demonstration of spike detection and spike sorting. a Time section of a differential raw trace (band-pass filtered 600-6000 Hz) from one electrode of a tetrode. Stimulus presentation is depicted in green. Red lines demonstrate the two manually set thresholds to detect spikes. b Results from a principal component analysis (PCA) after spike sorting which was based on the spike shape. In this example, eight spike clusters could be easily distinguished and classified as single units. c Spike shape limits plotted for each PCA cluster. Note that the spike shape monitored in each electrode of the tetrode is plotted. (TIF 8474 KB)

## Data Availability

MATLAB files containing the physiological data is accessible from the Figshare Repository: 10.6084/m9.figshare.22654084. Due to storage limitations, raw data including anatomical data can be made available upon request, Jerome Beetz (jerome.beetz@uni-wuerzburg.de).
