# Supplementary figures and images for: The influence of stimulus history on directional coding in the monarch butterfly brain
Source: J Comp Physiol A Neuroethol Sens Neural Behav Physiol. 2023 Apr 24;209(4):663–77. doi: 10.1007/s00359-023-01633-x (PMC10354184; doi:10.1007/s00359-023-01633-x)

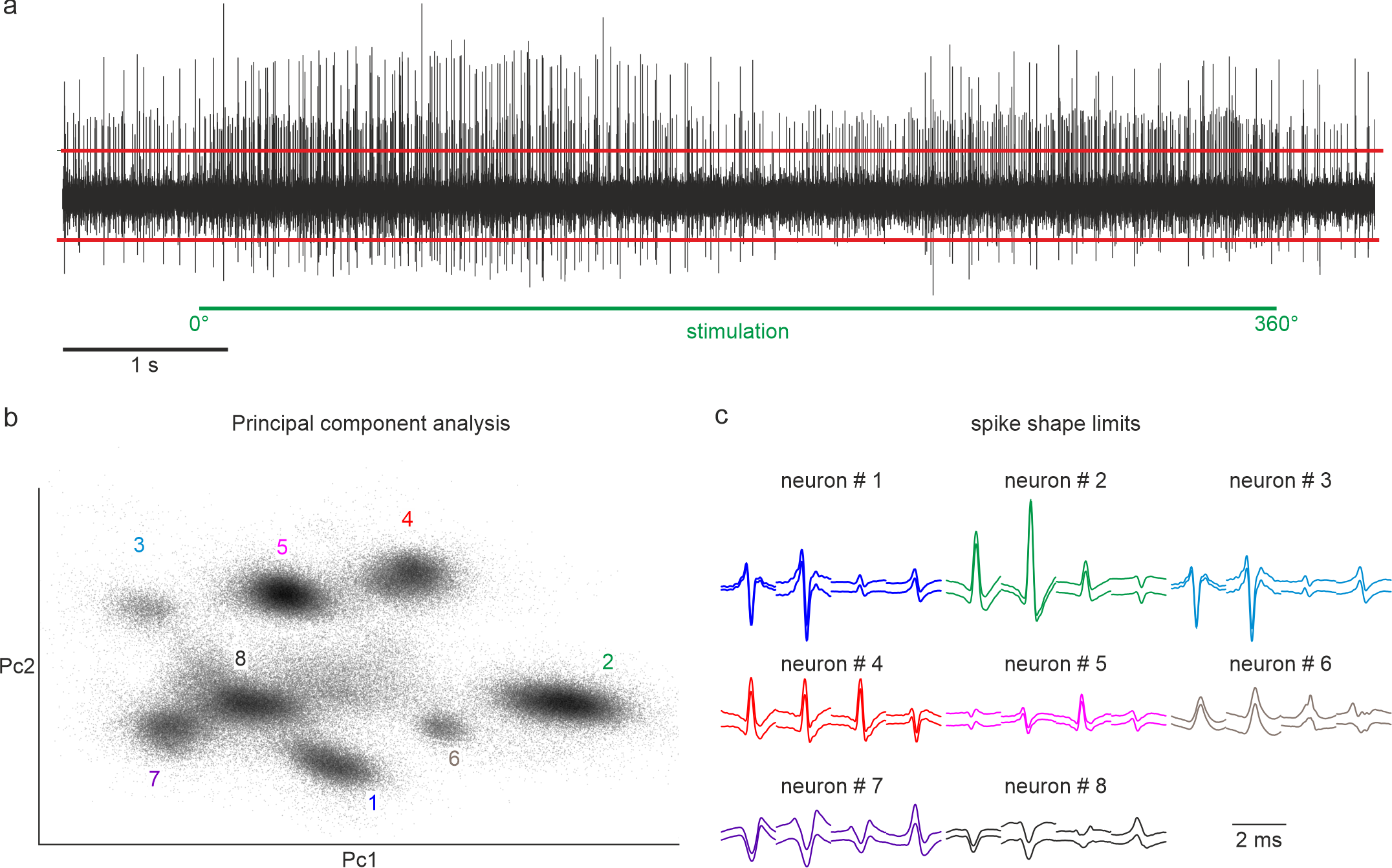

Supplement: Supplementary file 1 — Supplementary file1 Demonstration of spike detection and spike sorting. a Time section of a differential raw trace (band-pass filtered 600-6000 Hz) from one electrode of a tetrode. Stimulus presentation is depicted in green. Red lines demonstrate the two manually set thresholds to detect spikes. b Results from a principal component analysis (PCA) after spike sorting which was based on the spike shape. In this example, eight spike clusters could be easily distinguished and classified as single units. c Spike shape limits plotted for each PCA cluster. Note that the spike shape monitored in each electrode of the tetrode is plotted. (TIF 8474 KB) [file 359_2023_1633_MOESM1_ESM.tif]
